# Intracranial Aneurysm Predisposing to Terson’s Syndrome: Insights From a Systematic Review

**DOI:** 10.7759/cureus.84828

**Published:** 2025-05-26

**Authors:** Ali K Al-Shalchy, Ahmed S Al-Wassiti

**Affiliations:** 1 Department of Surgery, College of Medicine, University of Baghdad, Baghdad, IRQ

**Keywords:** aneurysm location, intraocular hemorrhage, subarachnoid hemorrhage, terson syndrome, vitrectomy

## Abstract

Terson syndrome (TS) is an intraocular hemorrhage associated with subarachnoid hemorrhage (SAH), often linked to specific intracranial aneurysm locations. This review and meta-analysis aim to explore the relationship between aneurysm location and the risk of developing TS.

A systematic search was conducted using PubMed and Scopus with the terms "Terson’s Syndrome" OR "Terson Syndrome." Studies involving adult patients in English were included. Data on aneurysm location, TS incidence, and clinical outcomes were extracted. The risk of bias was assessed using the Risk Of Bias In Non-randomized Studies of Interventions (ROBINS-I) tool and the Case Report (CARE) guidelines. Meta-analysis was performed using Microsoft Excel 2016 (Microsoft® Corp., Redmond, WA, USA).

Twenty-one studies with 201 patients were analyzed. Aneurysms in the posterior communicating artery (Pcom) were most frequently associated with TS (24.48%), followed by the anterior communicating artery (Acom) (23.87%). Endovascular coiling was the most common treatment (67.83%), with vitrectomy leading to improved visual outcomes. Aneurysms in the anterior circulation are strongly linked to TS. Early detection and timely surgical interventions, such as vitrectomy, are crucial for improving visual and clinical outcomes in patients with TS.

## Introduction and background

Terson syndrome (TS), first described by Moritz Litten in 1881 and later named by Albert Terson in 1900, refers to an intraocular hemorrhage associated with intracranial hemorrhage, most commonly subarachnoid hemorrhage (SAH)​ [1,2]. Although TS is frequently observed following SAH, its pathophysiology remains a subject of debate. The condition presents with various types of intraocular hemorrhages, including vitreous, preretinal, intraretinal, or subretinal bleeding​ [2]. Reported incidence varies widely, with estimates suggesting that 12.5% to 40% of patients with intracranial hemorrhage, particularly those with aneurysmal SAH, experience TS​ [3].

Diagnosing TS typically involves a combination of neuroimaging and ophthalmologic evaluation. While computed tomography (CT) and magnetic resonance imaging (MRI) can identify intracranial hemorrhages, direct visualization of intraocular bleeding is achieved through fundoscopy, ocular ultrasound, or optical coherence tomography (OCT), depending on resource availability and clinical urgency [1-3].

The risk of occurrence of TS has been related to higher clinical grades of SAH, such as Hunt and Hess (H&H) or Fisher grading scale, suggesting a relation between the severity of intracranial pressure (ICP) elevation and the likelihood of intraocular hemorrhage [3]. The H&H scale classifies the clinical severity of SAH based on neurological status, while the Fisher scale assesses the amount and distribution of blood on CT imaging; both are commonly used to predict outcomes and the likelihood of complications such as TS [3]. Aneurysm location has also been described as a predisposing factor, and several studies have linked anterior circulation aneurysms with TS, especially those involving the anterior communicating artery (Acom) [3].

In view of the clinical relevance of TS in SAH patients, it is important to study the possible anatomic predispositions that might influence its appearance. This systematic review is focused on studying the correlation between aneurysm characteristics and the risk of developing TS by presenting in depth the critical analysis of the literature and insights into the mechanisms underlying this complex syndrome.

## Review

Methods

This systematic review is performed using the Preferred Reporting Items for Systematic Reviews and Meta-Analyses (PRISMA) guidelines (2020) to ensure transparency and completeness of the process of study selection and analysis [4].

Search Strategy

An extensive literature search was conducted using two major databases, namely PubMed and Scopus. The search also included the use of the following terms: "Terson's Syndrome" OR "Terson Syndrome." The searching process was conducted in November 2024. No date limit was set to purposefully include all studies published up to this date.

Study Selection

Study selection was conducted using the Rayyan tool (Qatar Computing Research Institute, Qatar) for systematic reviews. The search results were imported into Rayyan, following which two reviewers screened the titles and abstracts independently. Studies were therefore included if they explored the exact relationship between the anatomical location of intracranial aneurysms and the occurrence of TS. The studies should involve adult patients ≥18 years of age and be written in English; hence, pediatric populations, non-human studies, and studies that did not provide adequate data on either the syndrome of Terson or aneurysm location should be excluded.

Data Extraction

Extracted data included author name, year of publication, study design, number of patients, demographics of patients, location of aneurysms, incidence of TS, and clinical outcomes. Individual study data were critically analyzed by two authors independently. Discrepancies were discussed among reviewers for consensus.

Results

**Figure 1:**
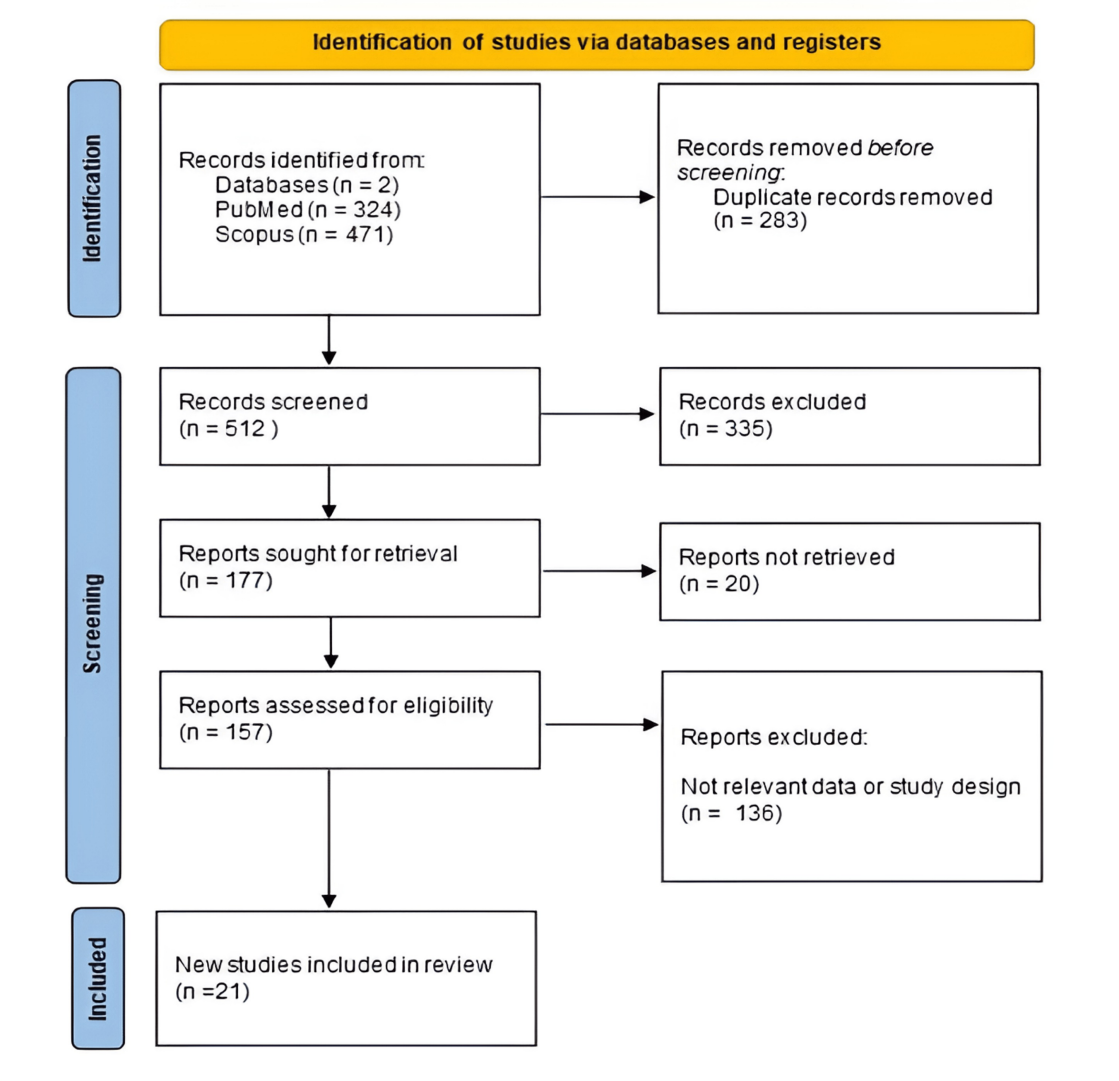
PRISMA Flowchart of the Included Studies PRISMA: Preferred Reporting Items for Systematic Reviews and Meta-Analyses

A total of 795 records were initially identified through database searches, including 324 records from PubMed and 471 from Scopus. After the removal of 283 duplicates, 512 records remained for screening based on titles and abstracts. Of these, 335 records were excluded for irrelevance, and 177 full-text reports were sought for retrieval. Twenty reports could not be accessed, leaving 157 articles for full-text assessment. The exclusion of 20 inaccessible reports may have introduced a degree of selection bias and could limit the completeness of the dataset. Following detailed eligibility screening, 136 articles were excluded due to unsuitable study design or lack of relevant data regarding aneurysm location and its association with TS. Ultimately, 21 studies met the inclusion criteria and were incorporated into the final systematic review and meta-analysis (Figure 1).

The quality of the included studies was assessed using the Case Report (CARE) guidelines (Table 1) [5] and the Risk Of Bias In Non-randomized Studies of Interventions (ROBINS-I) guidelines (Table 2) [6], and overall, the studies demonstrated good methodological quality, with minimal bias across key domains [7-27]. Accordingly, the interpretation of findings prioritized data from studies with higher methodological rigor, while results from lower-quality studies were considered with caution.

**Table 1:** Quality Assessment of Case Reports Using the CARE Guidelines CARE: Case Report

Study ID	First Author	Patient Information	Clinical Findings	Diagnostic Assessment	Therapeutic Interventions	Follow-Up Outcomes	Discussion/Conclusions	Overall Quality
1	Abduljabbar and Kahtani [7]	Not Comprehensive	Not Detailed	Thorough	Well-Documented	Reported	Relevant	Low
2	Inoue et al. [10]	Comprehensive	Detailed	Thorough	Well-Documented	Reported	Relevant	High
3	Issiaka et al. [11]	Comprehensive	Detailed	Thorough	Well-Documented	Reported	Relevant	High
4	Iwase and Tanaka [12]	Not Comprehensive	Not Detailed	Thorough	Well-Documented	Reported	Relevant	Moderate
5	Maistriau et al. [16]	Comprehensive	Detailed	Not Thorough	Well-Documented	Reported	Not Relevant	Moderate
6	Marrone et al. [17]	Comprehensive	Detailed	Thorough	Well-Documented	Reported	Relevant	High
7	Maslias et al. [18]	Comprehensive	Detailed	Thorough	Well-Documented	Reported	Relevant	High
8	Moynihan and Robinson [19]	Comprehensive	Detailed	Thorough	Well-Documented	Reported	Relevant	High
9	Murthy et al. [20]	Not Comprehensive	Not Detailed	Thorough	Well-Documented	Reported	Relevant	Moderate
10	Paquette et al. [21]	Not Comprehensive	Not Detailed	Not Thorough	Not Well-Documented	Reported	Relevant	Low
11	Phuyal et al. [22]	Comprehensive	Detailed	Thorough	Well-Documented	Reported	Relevant	High
12	Pinnola et al. [23]	Comprehensive	Detailed	Thorough	Not Well-Documented	Not Reported	Relevant	Moderate

**Table 2:** ROBINS-I Assessment of the Included Studies ROBINS-I: Risk Of Bias In Non-randomized Studies of Interventions

Study ID	Authors	Confounding	Selection of Patients	Classification of Interventions	Deviations From Intended Interventions	Missing Data	Measurement of Outcomes	Selection of Reported Results
1	Czorlich et al. [8]	Moderate	High	High	Moderate	Moderate	Low	Moderate
2	Göttsche et al. [9]	Moderate	High	High	Moderate	Moderate	Low	Moderate
3	Joswig et al. [13]	Low	High	High	Low	Moderate	Moderate	Moderate
4	Kang et al. [14]	Moderate	High	High	Moderate	Moderate	Moderate	Moderate
5	Lee et al. [15]	Moderate	High	High	Low	Moderate	Moderate	Moderate
6	Ramos-Estebanez et al. [24]	Moderate	High	High	Low	Moderate	Moderate	Moderate
7	Seif et al. [25]	Moderate	High	High	Low	Moderate	Moderate	Moderate
8	Stienen et al. [26]	Moderate	High	High	Low	Moderate	Moderate	Moderate
9	Wu et al. [27]	Moderate	High	High	Low	Moderate	Moderate	Moderate

A total of 21 studies were included in this systematic review and meta-analysis, with a cumulative sample size of 201 patients (Table 3). The search was performed on September 28, 2024, to ensure the inclusion of all relevant studies published up to that date.

**Table 3:** Studies Investigating Intracranial Aneurysm Locations and Terson Syndrome (TS) Outcomes Acom: Anterior communicating artery; SAH: Subarachnoid hemorrhage; ICH: Intracerebral hemorrhage; ICA: Internal carotid artery; MCA: Middle cerebral artery; N/A: Not available

Study ID	First Author	Year	Country	Study Design	Sample Size	Population Characteristics	ICH Type	Aneurysm Location	Aneurysm Size
1	Abduljabbar and Kahtani [7]	1992	KSA	Case Report	1	A 40-year-old woman with hypertension	SAH	Acom	N/A
2	Czorlich et al. [8]	2016	Germany	Retrospective	16	Ages 18-87 (mean 53.8)	SAH	Anterior circulation common, no link to TS	No link
3	Göttsche et al. [9]	2023	Germany	Retrospective	78	71.8% females, mean age 52.8	SAH	Anterior circulation (37.2%)	No link
4	Inoue et al. [10]	2006	Japan	Case Report	1	A 61-year-old male with uncontrolled HT	SAH	Vertebral artery	N/A
5	Issiaka et al. [11]	2022	Morocco	Case Report	1	A 55-year-old diabetic hypertensive man	SAH	Internal carotid	N/A
6	Iwase and Tanaka [12]	2006	Japan	Case Report	1	A 41-year-old male with visual loss	SAH	Left ICA aneurysm	N/A
7	Joswig et al. [13]	2016	Switzerland	Prospective	6	Ages 45-69, mostly female	SAH	Acom, A1/A2, ICA	33-50% small, 16% large
8	Kang et al. [14]	2020	South Korea	Retrospective	31	80% female, ages 35-73	SAH	MCA (40%), Acom (30%)	N/A
9	Lee et al. [15]	2015	South Korea	Retrospective	322	Mean age 54.3	SAH	N/A	Larger aneurysms linked to TS
10	Maistriau et al. [16]	2015	Belgium	Case Report	1	A 39-year-old female with hypertension	SAH	Acom	N/A
11	Marrone et al. [17]	2024	Italy	Case Report	1	A 52-year-old female with TS	SAH	MCA	6 × 4 mm
12	Maslias et al. [18]	2023	Switzerland	Case Report	2	63 and 47 years old females	SAH	Acom	4.5-6 mm
13	Moynihan and Robinson [19]	2012	Australia	Case Report	1	A 50-year-old male	SAH/SDH	Left ACA and ICA	6 × 3 × 2 mm
14	Murthy et al. [20]	2002	UK	Case Report	1	A 38-year-old male with bilateral TS	SAH	Left carotid-ophthalmic	N/A
15	Paquette et al. [21]	2010	Canada	Case Report	1	A 51-year-old male with TS	SAH	Acom	3 mm
16	Phuyal et al. [22]	2024	Nepal	Case Report	1	A 48-year-old female with TS	SAH	Left vertebral artery	N/A
17	Pinnola et al. [23]	1998	Brazil	Case Report	1	A 30-year-old female with seizures and vision loss	SAH	Right MCA	N/A
18	Ramos-Estebanez et al. [24]	2018	USA	Pilot Study	79	Mean age 54.7, 85% female	SAH	Basilar (42.9%)	6.2 mm
19	Seif et al. [25]	2014	Canada	Prospective	10	Mean age 61.6, 60% with hypertension	SAH	Acom (50%)	N/A
20	Stienen et al. [26]	2012	Germany	Prospective	60	Mean age 54.12	SAH	Acom, Pcom	N/A
21	Wu et al. [27]	2012	China	Case Series	16	11 females, 5 males	SAH	Anterior circulation in 15/16	N/A

Treatment Modalities and Patient Outcomes

Among the 21 included studies, treatment modalities included surgical clipping, endovascular coiling, vitrectomy, flow diversion, and conservative management. Coiling was the most frequently utilized approach. Surgical clipping was employed in patients with larger or complex aneurysms or where immediate decompression was required (Table 4).

**Table 4:** Treatment Strategy for Terson Syndrome (TS) Patients aSAH: Aneurysmal subarachnoid hemorrhage; SAH: Subarachnoid hemorrhage; ICP: Intracranial pressure; GCS: Glasgow Coma Scale; H&H: Hunt and Hess; mRS: Modified Rankin Scale; BCVA: best-corrected visual acuity; PPV: pars plana vitrectomy; WFNS: World Federation of Neurosurgical Societies; N/A: Not available; MCS: minimally conscious state

Study ID	First Author	Treatment	Time to Intervention (Days)	Prognostic Outcomes	Outcome Measures	Mortality Rate (%)	Neurological Outcome	Visual Outcome	Follow-Up Length
1	Abduljabbar and Kahtani [7]	Surgery	1	TS linked to poor outcomes	Worse GCS & H&H	100	Initial vision improvement, then rebleeding	Improved vision, then worsened	3 days
2	Czorlich et al. [8]	No link to treatment	N/A	Worse outcomes with high ICP & unconsciousness	Higher ICP, higher mortality	N/A	Worse neurological outcomes (lower GCS, H&H)	N/A	5 years
3	Göttsche et al. [9]	Visual improvement after surgery	42-54	Higher WFNS grades, seizures	Higher WFNS, seizures, visual recovery	N/A	Worse neurological outcomes	Significant visual improvement	N/A
4	Inoue et al. [10]	Conservative	N/A	Recovered, but rerupture led to death	Significant decline after rerupture	100	Initial recovery, but death later	Severe visual loss	14 days
5	Issiaka et al. [11]	Embolization	2	Good visual and neurological outcomes	Full recovery	0	Full neurological recovery	Good visual recovery	1.5 years
6	Iwase and Tanaka [12]	Surgery	90	Visual recovery post-surgery	Significant visual improvement	0	Full neurological recovery	Significant visual recovery	6 months
7	Joswig et al. [13]	Coiling (67%)	N/A	High ICP linked to worse outcomes	High ICP linked to worse outcomes	83	Poor neurological outcomes	No visual details	3 months
8	Kang et al. [14]	N/A	N/A	Worse GCS and H&H scores	Higher H&H linked to TS	0	Worse neurological outcomes	No significant visual impairment	9.5 months
9	Lee et al. [15]	Coiling more common in TS	N/A	TS linked to worse outcomes, higher mortality	Higher WFNS grades, larger aneurysms linked to TS	N/A	Worse neurological outcomes	No visual details	N/A
10	Maistriau et al. [16]	Surgery	N/A	Vision improved post-surgery, patient died of infection	Poor visual outcomes without early surgery	0	Poor neurological recovery	Vision improved but patient died	N/A
11	Marrone et al. [17]	Coiling	N/A	Significant visual recovery	Significant visual recovery	0	Full neurological recovery	Significant visual recovery	1 week
12	Maslias et al. [18]	Surgery	1	Delayed diagnosis led to poor outcome in one case	Full recovery for one patient, MCS for the other	0	Neurological deficits in one patient	Visual recovery in both	N/A
13	Moynihan and Robinson [19]	Coiling	2	Persistent visual impairment	Poor visual recovery	0	No neurological deficits	Visual acuity remained poor	15 days
14	Murthy et al. [20]	Surgery	3	Significant visual recovery post-surgery	Vision improved to 6/6 in one eye	0	Full neurological recovery	Significant vision improvement	2 months
15	Paquette et al. [21]	Conservative	N/A	Gradual visual recovery	Vision recovered with conservative treatment	0	Neurologically intact	Gradual visual recovery	6 months
16	Phuyal et al. [22]	Flow diverter	7	Full vision recovery after treatment	Full vision recovery	0	Full neurological recovery	Full vision recovery	21 days
17	Pinnola et al. [23]	Surgery	N/A	Vision improved post-surgery	Neurological recovery favorable	0	Favorable neurological recovery	Vision improved	6 months
18	Ramos-Estebanez et al. [24]	Surgery	N/A	TS detected in 22.6%, visual improvement common	Significant visual improvement	N/A	Visual recovery in most patients	Significant visual recovery	6 weeks
19	Seif et al. [25]	N/A	N/A	Worse outcomes in TS patients	Higher mortality, worse GCS & H&H	N/A	Worse neurological outcomes	No visual recovery data	1 year
20	Stienen et al. [26]	Surgery	N/A	TS linked to worse outcomes	Higher mortality, worse neurological outcomes	36.4	Poor functional outcomes	No visual data	6-12 months
21	Wu et al. [27]	Conservative	N/A	TS in 14.16% of aSAH cases	Higher mortality, worse outcomes	12.5	Worse neurological outcomes	Vision improved with conservative treatment	6 months

Time to intervention varied, with several cases receiving surgical treatment within the first one to three days post-hemorrhage, correlating with better visual and neurological outcomes. Delays in intervention, as observed in cases such as Iwase and Tanaka (90 days) [12], were associated with an increased risk of complications and, in some instances, fatal outcomes.

Neurologically, TS was associated with worse clinical grades at presentation. Higher H&H and World Federation of Neurosurgical Societies (WFNS) scores, alongside lower Glasgow Coma Scale (GCS) values, were common findings in TS cohorts. Visual outcomes were heterogeneous across studies. Vitrectomy yielded the most consistent visual improvement, with several cases documenting substantial recovery in best-corrected visual acuity (BCVA). For example, visual acuity improved from light perception or counting fingers to 6/6 postoperatively in some patients. In contrast, conservative management often resulted in partial or no recovery of visual function.

Clinical and Neurological Outcomes

Patients with TS were generally associated with worse clinical outcomes compared to those without TS. Higher grades on the GCS and H&H scale were observed in TS patients, indicating more severe neurological impairment at presentation. Furthermore, patients with TS exhibited a higher mortality rate.

Visual Outcomes

Visual outcomes varied depending on the intervention. In patients undergoing vitrectomy, significant improvement in visual acuity has been observed in certain case studies. For instance, in a cohort of patients who underwent vitrectomy, the preoperative BCVA improved from a mean of 0.03 (± 0.08) to 0.76 (± 0.21) postoperatively, indicating a substantial enhancement in visual function. However, visual recovery was less pronounced in patients who were managed conservatively or who experienced delays in surgical intervention, such as vitrectomy.

Discussion

TS represents one potentially severe complication of an SAH, characterized by increased ICP, most often associated with aneurysms of the anterior circulation. When the aneurysm is dealing with extremely distal sites - for instance, the Acom - the risk not only significantly goes up but increases, leading to poor neurological outcomes and even retinal hemorrhages [28,29]. This review thus represents a systematic review of the association between intracranial aneurysm and the occurrence or development of TS in patients with SAH.

In fact, this review showed that anterior circulation aneurysms, particularly in the Acom, are related to TS. Notable differences in the anatomical proximity of ruptured cerebral aneurysms to the optic nerve and optic chiasm may provide insights into the pathophysiology of TS. Acom aneurysms are anatomically closer to the optic chiasm, which may predispose patients to visual disturbances due to direct compression or ischemia following rupture. In contrast, posterior communicating artery (Pcom) aneurysms are in close proximity to the optic nerve and can cause localized optic nerve compression or vascular compromise, potentially affecting venous drainage from the retina. These positional differences may explain the variable incidence and severity of intraocular hemorrhage observed in patients with TS. Furthermore, the disruption of venous outflow from the retina due to elevated ICP or vascular compression by aneurysms may contribute to retinal hemorrhages characteristic of TS [30]. The data indicated that TS was more prevalent among females, as only 28.28% of patients were male. The higher prevalence of TS in females may reflect anatomical or hormonal influences on vascular reactivity and venous drainage, or it may be influenced by reporting bias due to increased health-seeking behavior among females; however, further studies are needed to clarify this disparity.

As for the treatment, most aneurysmal SAHs in patients with TS were treated with endovascular coiling, followed by surgical clipping. While 67.83% of cases were treated with endovascular coiling and 31.16% with surgical clipping, the prognosis varied significantly between the two groups. Studies suggest coiling is associated with better visual outcomes, whereas clipping may be preferred in larger aneurysms requiring complete exclusion of the lesion. However, further analysis is warranted to control for factors such as aneurysm size, location, and patient comorbidities. These findings were supported by Lee et al. (2015) [15], who reported that the incidence of TS was higher in coiling patients than in clipping patients, with p < 0.001.

Among patients with TS, the visual outcome after vitrectomy, in particular, was very good, and indeed, an improvement in several studies was stated. An example includes the improvement in Göttsche et al. (2023) [9], where a great enhancement in visual acuity was noted, following the vitrectomy carried out from BCVA 0.03 to 0.76. Indeed, Iwase and Tanaka (2006) documented a remarkable visual recovery following surgery in a case study. This highlights that timely ophthalmologic intervention is essential for preserving the optimum possible vision in patients with TS. Ju et al. (2020) [29] found that early vitrectomy in patients with TS can lead to rapid and optimal visual recovery, significantly reducing the incidence of complications, which underscores the importance of prompt surgical intervention to enhance visual outcomes. In general, neurological presentations were always worse in patients with TS throughout the reviewed studies. The choice between endovascular treatment and direct surgery for cerebral aneurysms is often guided by factors such as aneurysm morphology, patient comorbidities, and surgical risk. However, when considering the prevention of TS, direct surgical clipping may offer advantages in patients at high risk. Surgical clipping provides immediate access to decompress the intracranial compartment, allowing better control of ICP. In contrast, endovascular coiling, while minimally invasive, may not address the rapid ICP fluctuations that could contribute to the development of intraocular hemorrhages characteristic of TS [29].

This review brought to light the importance of early diagnosis and intervention in patients with TS. In addition, the remarkable improvements in visual acuity following vitrectomy underscore the importance of referring patients presenting with TS for urgent ophthalmologic evaluation and treatment.

Limitations

There are some limitations to the findings of this review. A considerable number of the included studies reported small sample sizes, especially most case reports, which limit the generalization of results. Moreover, while pooled data provide useful information, heterogeneity in the nature of included studies pertaining to population characteristics, treatment modalities, and outcome measures could affect the results. Further studies are needed using larger cohorts, including standardized outcome reporting, to further elucidate the relationship between aneurysm location and the development of TS.

## Conclusions

This review highlights a strong link between anterior circulation aneurysms, particularly in the Acom and Pcom, and the development of TS in SAH patients. Early detection and timely interventions, such as vitrectomy, significantly improve visual outcome, especially in female patients, who show higher TS prevalence and worse prognosis as observed in this review.

Future research should focus on understanding the anatomical and hemodynamic factors behind this association, exploring gender-specific risks, and utilizing advanced diagnostic tools like OCT for early TS detection. Large-scale studies on long-term outcomes and refined treatment protocols will further improve care for SAH patients with TS.

## References

[1] Hayreh SS (2022). Pathogenesis of Terson syndrome. Indian J Ophthalmol.

[2] Lima-Fontes M, Leuzinger-Dias M, Rodrigues R (2023). Terson syndrome - clinical presentation, management, and visual outcomes in a tertiary centre. Clin Ophthalmol.

[3] Kumaria A, Gruener AM, Dow GR, Smith SJ, Macarthur DC, Ingale HA (2022). An explanation for Terson syndrome at last: the glymphatic reflux theory. J Neurol.

[4] Page MJ, McKenzie JE, Bossuyt PM (2021). The PRISMA 2020 statement: an updated guideline for reporting systematic reviews. BMJ.

[5] Sterne JA, Hernán MA, Reeves BC (2016). ROBINS-I: a tool for assessing risk of bias in non-randomised studies of interventions. BMJ.

[6] Gagnier JJ, Kienle G, Altman DG, Moher D, Sox H, Riley D (2013). The CARE guidelines: consensus-based clinical case report guideline development. J Diet Suppl.

[7] Abduljabbar M, Kahtani ES (1992). Terson's syndrome in a Saudi patient: a case report. Ann Saudi Med.

[8] Czorlich P, Skevas C, Knospe V, Vettorazzi E, Westphal M, Regelsberger J (2016). Terson's syndrome - pathophysiologic considerations of an underestimated concomitant disease in aneurysmal subarachnoid hemorrhage. J Clin Neurosci.

[9] Göttsche J, Knospe V, Sauvigny T (2023). Terson syndrome in patients with aneurysmal subarachnoid hemorrhage: a 10-year single-center experience. Neurocrit Care.

[10] Inoue T, Tsutsumi K, Shigeeda T (2006). Terson's syndrome as the initial symptom of subarachnoid hemorrhage caused by ruptured vertebral artery aneurysm. Case report. Neurol Med Chir (Tokyo).

[11] Issiaka M, Mchachi A, Rachid R, Belhadji ME, Mahazou I, Banao M (2022). Terson syndrome: two case reports. Int J Surg Case Rep.

[12] Iwase T, Tanaka N (2006). Bilateral subretinal haemorrhage with Terson's syndrome. Graefes Arch Clin Exp Ophthalmol.

[13] Joswig H, Epprecht L, Valmaggia C, Leschka S, Hildebrandt G, Fournier JY, Stienen MN (2016). Terson syndrome in aneurysmal subarachnoid hemorrhage-its relation to intracranial pressure, admission factors, and clinical outcome. Acta Neurochir (Wien).

[14] Kang HM, Cho JM, Kim SY, Choi JH (2020). Clinical characteristics of asymptomatic Terson syndrome in the patients with aneurysmal subarachnoid hemorrhage. Int J Ophthalmol.

[15] Lee GI, Choi KS, Han MH, Byoun HS, Yi HJ, Lee BR (2015). Practical incidence and risk factors of Terson’s syndrome: a retrospective analysis in 322 consecutive patients with aneurysmal subarachnoid hemorrhage. J Cerebrovasc Endovasc Neurosurg.

[16] Maistriau C, Duprez T, Hantson P (2016). Terson's syndrome in aneurysmal subarachnoid haemorrhage. Acta Neurol Belg.

[17] Marrone S, Pizzo C, Paolini F (2024). Atypical Terson syndrome after subarachnoid hemorrhage from middle cerebral artery aneurysm rupture during coitus. Surg Neurol Int.

[18] Maslias E, Vijiala S, Epiney JB, Konstantinidis L, Kawasaki A, Diserens K (2023). Terson syndrome: not to be missed in patients with disorders of consciousness. Brain Sci.

[19] Moynihan G, Robinson K (2012). Terson's syndrome: subarachnoid haemorrhage presenting as sudden visual loss. Emerg Med Australas.

[20] Murthy S, Salas D, Hirekataur S, Ram R (2002). Terson's syndrome presenting as an ophthalmic emergency. Acta Ophthalmol Scand.

[21] Paquette F, Darsaut TE, Sebag M, Weill A (2010). Terson's syndrome. Can J Neurol Sci.

[22] Phuyal P, Chhetri ST, Khanal D, Phuyal S, Paudel S, Hamal D, Regmi B (2024). Terson syndrome in association with sub-arachnoid hemorrhage: a case report. Ann Med Surg (Lond).

[23] Pinnola GC, Corrêa SM, Ribeiro SB, Leboreiro-Fernandez A, Marquez JO (1998). Terson's syndrome. Report of a case with favorable outcome. Arq Neuropsiquiatr.

[24] Ramos-Estebanez C, Kohen M, Pace J (2019). Bedside optical coherence tomography for Terson's syndrome screening in acute subarachnoid hemorrhage: a pilot study. J Neurosurg.

[25] Seif GI, Teichman JC, Reddy K, Martin C, Rodriguez AR (2014). Incidence, morbidity, and mortality of Terson syndrome in Hamilton, Ontario. Can J Neurol Sci.

[26] Stienen MN, Lücke S, Gautschi OP, Harders A (2012). Terson haemorrhage in patients suffering aneurysmal subarachnoid haemorrhage: a prospective analysis of 60 consecutive patients. Clin Neurol Neurosurg.

[27] Wu LN, He T, Xing YQ, Shen Y (2013). Incidence of Terson's syndrome in patients with SAH in a Chinese hospital. Curr Eye Res.

[28] Bhende P, Maitra P (2023). Anatomical and surgical considerations and outcomes in infantile Terson syndrome. Indian J Ophthalmol.

[29] Ju C, Li S, Huang C, Li Y, Kyungwan H, Zhou F, Li J (2020). Clinical observations and considerations in the treatment of Terson syndrome using 23G vitrectomy. Int Ophthalmol.

[30] Durst CR, Starke RM, Gaughen J, Nguyen Q, Patrie J, Jensen ME, Evans AJ (2014). Vision outcomes and major complications after endovascular coil embolization of ophthalmic segment aneurysms. AJNR Am J Neuroradiol.

